# Clinical Clearance of Actinic Keratoses with Tirbanibulin 1% Ointment: Evidence from a Meta-Analysis

**DOI:** 10.3390/life16081330

**Published:** 2026-08-13

**Authors:** Mircea Tampa, Clara Matei, Madalina Irina Mitran, Cristina Iulia Mitran, Weeratian Tawanwongsri, Paula Loredana Chiriac, Ecaterina Rinja, Corina Daniela Ene, Ilinca Nicolae, Milena Tocut, Simona Roxana Georgescu

**Affiliations:** 1 Department of Dermatology, ‘Carol Davila’ University of Medicine and Pharmacy, 020021 Bucharest, Romania; mircea.tampa@umfcd.ro (M.T.); clara.matei@umfcd.ro (C.M.); paula-loredana.chiriac@drd.umfcd.ro (P.L.C.); simona.georgescu@umfcd.ro (S.R.G.); 2 Department of Dermatology, ‘Victor Babes’ Clinical Hospital for Infectious Diseases, 030303 Bucharest, Romania; drnicolaei@yahoo.ro; 3 Department of Microbiology, ‘Carol Davila’ University of Medicine and Pharmacy, 020021 Bucharest, Romania; 4 Division of Dermatology, Department of Internal Medicine, School of Medicine, Walailak University, Nakhon Si Thammarat 80160, Thailand; weeratian.ta@wu.ac.th; 5 Gastroenterology Department, Clinical Emergency Hospital of Bucharest, 014461 Bucharest, Romania; ecaterina.rinja@gmail.com; 6 Department of Nephrology, ‘Carol Davila’ University of Medicine and Pharmacy, 020021 Bucharest, Romania; corina.ene@umfcd.ro; 7 Department of Nephrology, ‘Carol Davila’ Nephrology Hospital, 010731 Bucharest, Romania; 8 Israel Faculty of Medicine, Tel Aviv University, Tel Aviv 69978, Israel; milena.tocut@gmail.com; 9 Internal Medicine C Department, Wolfson Medical Center, Holon 58100, Israel

**Keywords:** actinic keratosis, tirbanibulin, meta-analysis

## Abstract

Actinic keratoses (AKs) are skin conditions that arise from the proliferation of atypical keratinocytes and are considered precursors to cutaneous squamous cell carcinoma. There are several treatment options available, which can be categorized into two main groups: lesion-directed therapies and field-directed therapies. Tirbanibulin 1% ointment has emerged as a new treatment for AKs. We conducted a meta-analysis to evaluate the effectiveness of tirbanibulin by analyzing the rates of complete clearance (CC) and partial clearance (PC) in randomized controlled trials (RCTs) and real-world studies. Our search included four databases: PubMed, Google Scholar, Scopus, and Web of Science. After applying specific inclusion and exclusion criteria, we identified 11 study cohorts from 10 publications. The CC rate was reported in 10 of the 11 studies, involving a total of 2092 patients, with 874 CC events observed. This resulted in an overall pooled CC rate of 52.05% (95% CI: 42.43–61.52%). The PC rate was also reported in 10 of the 11 studies, involving a total of 2367 patients, with 1631 PC events recorded. This led to an overall pooled PC rate of 71.96% (95% CI: 64.89–78.09%). In conclusion, tirbanibulin 1% ointment has shown clinical effectiveness and should be considered a promising treatment for AKs.

## 1. Introduction

Actinic keratoses (AKs) are common lesions of the skin characterized by the aberrant proliferation of dysplastic epidermal keratinocytes [1].

The pathogenesis of AKs remains a topic of debate within the scientific community. Some researchers propose that AKs serve as precursors to cutaneous squamous cell carcinoma (cSCC), while others consider them a form of cSCC in situ. The progression rates from AK to cSCC can vary widely, ranging from 0.0015% to 16% [2]. This event underscores the need to treat these lesions adequately. Current therapeutic options for AKs can be divided into two main classes: lesion-directed therapies (cryotherapy, laser ablation, and curettage) and field-directed therapies (photodynamic therapy and topical agents such as imiquimod, 5-fluorouracil, and diclofenac sodium) [3,4].

In 2020, the Food and Drug Administration (FDA) approved tirbanibulin 1% ointment for the treatment of Olsen grade I AKs on the scalp and face as a field-directed therapy (clinically, AKs are classified into three categories (I-III) based on their thickness and hyperkeratosis according to the Olsen scale). One year later, it received approval from the European Medicines Agency (EMA) [5]. Tirbanibulin is a synthetic microtubule inhibitor that blocks tubulin polymerization and alters Src kinase signaling. The approved treatment protocol for AKs consists of applying the ointment topically for 5 consecutive days over an area of up to 25 cm^2^ [6]. Numerous studies have demonstrated the effectiveness of tirbanibulin 1% ointment in treating AK so far. However, a comprehensive synthesis of its efficacy regarding complete and partial clearance rates remains limited. Previous meta-analyses included relatively few studies involving small groups of patients, with real-world evidence remaining particularly scarce. Our aim was to assess the effectiveness of tirbanibulin 1% ointment for AK treatment through the inclusion of the latest real-world studies with larger patient populations.

## 2. Materials and Methods

We performed a meta-analysis following the Preferred Reporting Items for Systematic Reviews and Meta-Analyses (PRISMA) guidelines.

### 2.1. The Search Strategy

The search strategy was conducted by browsing four databases: PubMed (MEDLINE), Scopus, Web of Science, and Google Scholar (from database inception to 28 May 2026). There were no restrictions on the language or period of publication. We used MeSH terms and free keywords, including (“tirbanibulin” OR “Klisyri”) AND (“actinic keratosis” OR “solar keratosis”). The complete database-specific search strategies are presented in Supplementary Materials.

Additionally, we manually reviewed articles included in existing systematic reviews and dermatological guidelines. The references were imported into Zotero, where we identified and removed duplicates. The Rayyan platform (Rayyan Systems Inc., Cambridge, MA, USA) was utilized for reference screening. Two reviewers independently made the inclusion or exclusion decisions for references based on pre-established criteria, without the use of artificial intelligence tools.

### 2.2. Eligibility Criteria

We included studies that met the following criteria: randomized controlled trials (RCTs) and real-world studies involving adult patients (≥18 years) diagnosed with AKs (based on clinical features) located on the face or scalp and treated with tirbanibulin 1% ointment administered topically once daily for 5 consecutive days. The primary efficacy endpoint was the percentage of patients with complete clearance (CC), defined as a 100% reduction in lesions on the treated area, while the secondary endpoint was the percentage of patients with partial clearance (PC), defined as a reduction of ≥75% in lesions on the treated area, assessed approximately 8 weeks after treatment. Only studies that included at least 10 participants were analyzed. The studies used vehicle control (placebo)/other topical therapies or did not include a control group for comparison.

Animal studies, case series, case reports, review articles, editorials, conference papers, and studies that analyzed patients with AKs in other locations (e.g., arms, legs), as well as studies that used concentrations or therapeutic regimens different from the approved protocol, were excluded.

### 2.3. Data Extraction

The data were independently extracted by two reviewers (M.T. and C.I.M.) using a standardized form. Any inconsistencies were discussed and resolved by a third reviewer (M.I.M.). For each study, the following data were collected: lead author, year of publication, type of study (RCT or real-world study), total number of participants, number of patients achieving CC, number of patients achieving PC at the time closest to eight weeks, participant characteristics (mean age, sex), AK grade, and follow-up period.

Most trials used a cumulative approach, meaning that the counts of partial clearance (PC) already included patients who later achieved complete clearance (CC). In the few studies that separated these two categories, we combined the CC responders with the PC group. This ensured consistent calculation of success rates across all studies.

### 2.4. Statistical Analysis

Statistical analysis was performed using R software (version 4.6.0 titled ‘Because it was there’; R Foundation for Statistical Computing, Vienna, Austria) utilizing the ‘meta’ package. The pooled proportions for complete and partial clearance rates were derived from absolute numbers (number of events and total sample sizes) using the metaprop function. The inverse variance method was employed to weigh individual trials and calculate the pooled estimates with 95% confidence intervals (CIs). The heterogeneity of the studies was assessed and quantified by the Cochrane Q chi-squared test and I^2^ statistics. The heterogeneity was considered substantial when the I^2^ was greater than 50%. Given the variability of the studies, we used a random-effects model. Statistical significance was defined as a *p* value < 0.05. Publication bias was assessed by the Egger and Begg tests. In addition, we performed a leave-one-out sensitivity analysis. Finally, a critical back-transformation was performed to re-map the integrated summary estimates and their 95% CIs from the logit domain to the conventional linear scale using the inverse logistic function: p = e^logit(p)/(1 + e^logit(p)).

The included randomized studies were evaluated using the revised Cochrane Risk of Bias tool for randomized trials (RoB 2), focusing on bias arising from the randomization process, deviations from intended interventions, missing outcome data, measurement of the outcome, and selection of the reported results. Nonrandomized studies were assessed using the Joanna Briggs Institute Critical Appraisal Checklist for Case Series. This checklist was selected because, for the outcomes included in this meta-analysis, these studies contributed uncontrolled single-arm patient-level clearance data without a concurrent comparator, irrespective of the design terminology used in the original publications. The checklist covered participant selection, measurement and identification of the condition, consecutive and complete inclusion, reporting of demographic and clinical information, outcome reporting, site information, and statistical analysis. Judgments were summarized descriptively for each domain and overall.

## 3. Results

**Figure 1:**
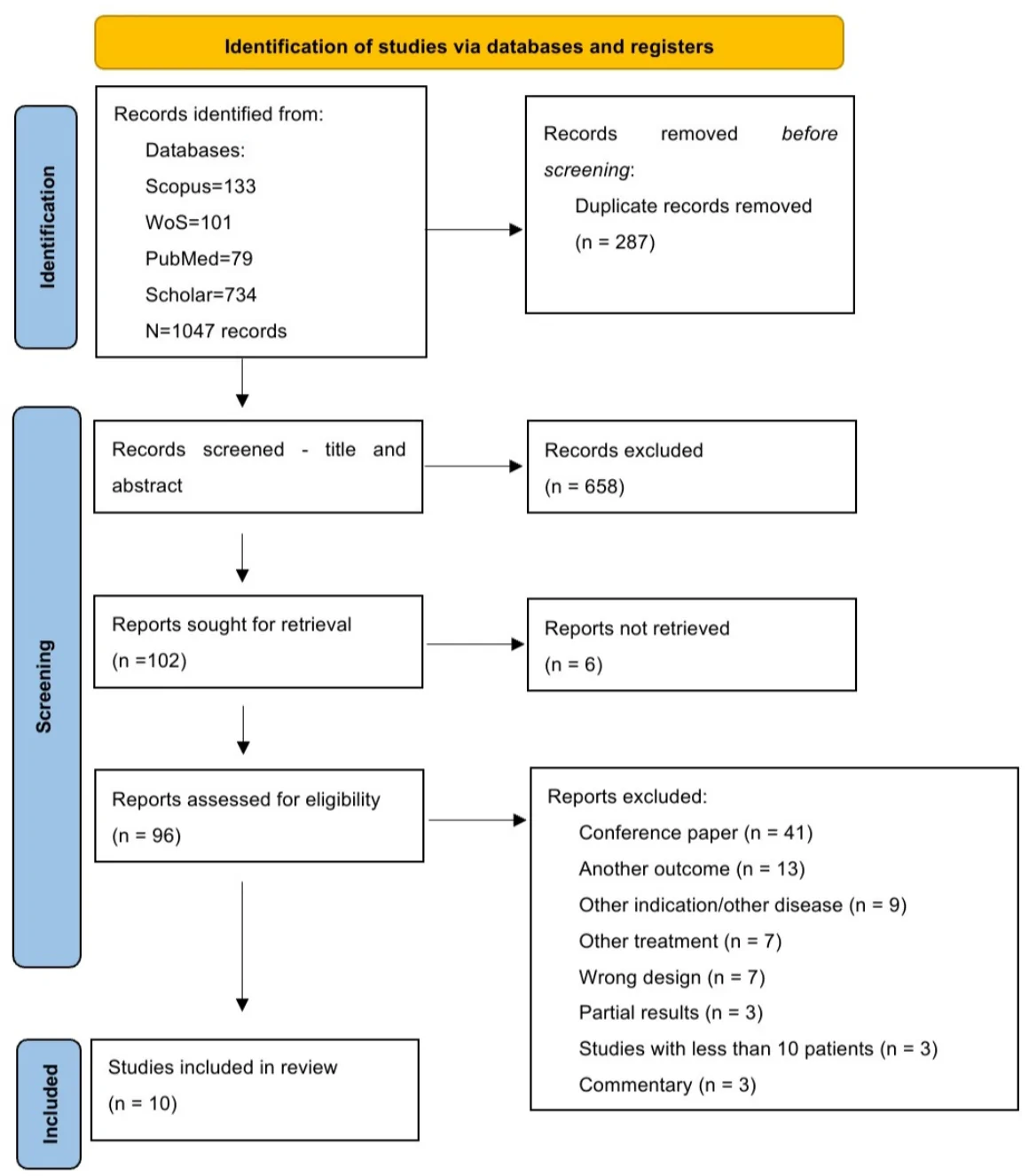
PRISMA 2020 flow diagram of the study [7].

Following the search strategy, we identified a total of 1047 articles (Figure 1). We identified and removed 287 duplicates, resulting in 760 records for screening. For the final evaluation stage, 102 articles were selected for full-text assessment. Of these, 6 articles could not be retrieved due to the unavailability of the full text, while the remaining articles underwent a rigorous evaluation process in Rayyan. Subsequently, 10 articles met the eligibility criteria for inclusion in the analysis, with individual study populations ranging from 15 to 679 patients. In the article by Blauvelt et al., two trials are presented that will be evaluated separately in this meta-analysis. Consequently, we examined 11 studies.

Table 1 summarizes the characteristics of the patients included in the studies. In many studies, data regarding patients’ immune status were not available.

**Figure 2:**
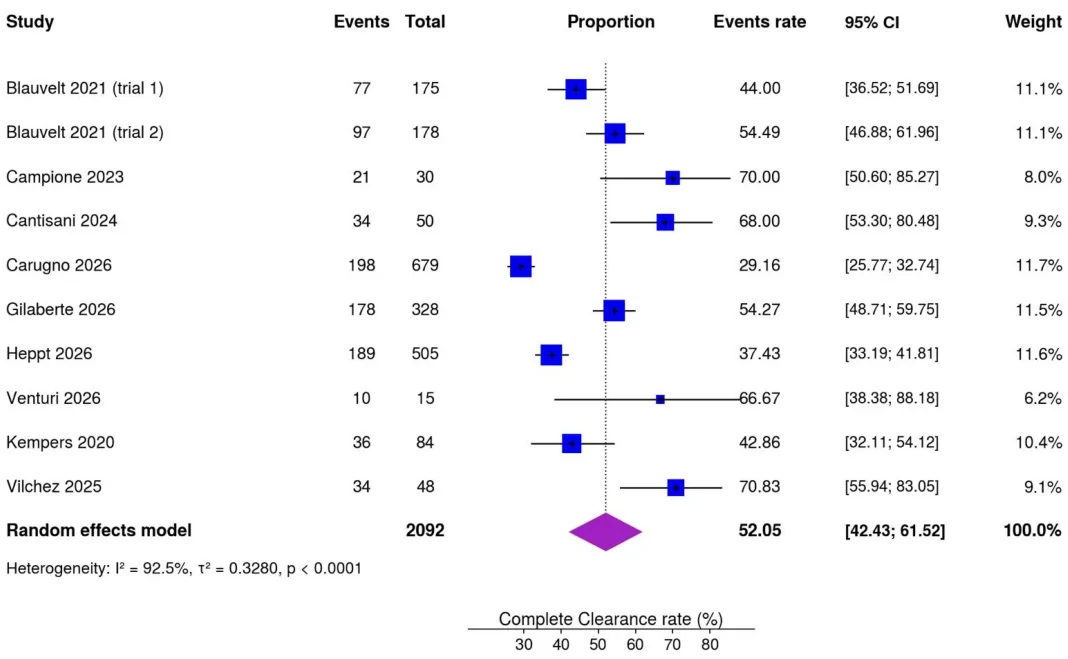
Complete clearance rates across the included studies [8,9,10,11,12,13,14,16,17].

The CC rate was reported in 10 of the 11 studies included, encompassing a total of 2092 patients. A total of 874 CC events were reported, resulting in an overall pooled CC rate of 52.05% (95% CI: 42.43–61.52%) (Figure 2).

**Figure 3:**
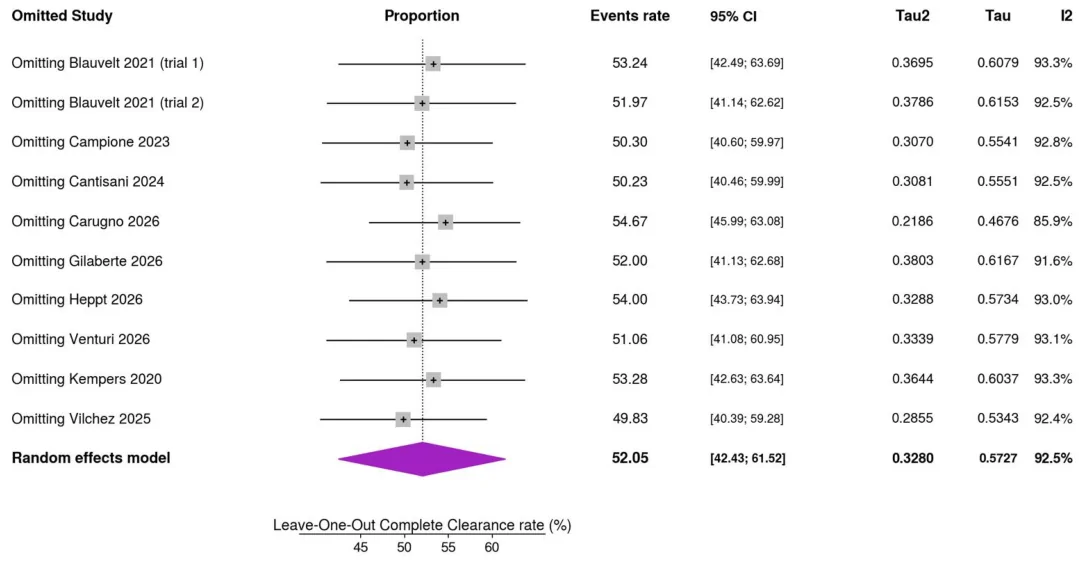
Leave-one-out sensitivity analysis for complete clearance rate [8,9,10,11,12,13,14,16,17].

The leave-one-out sensitivity analyses indicated that the estimated CC rate was stable, consistently falling within a narrow range of 49.83% to 54.67% after successively excluding individual studies (Figure 3). None of the studies significantly influenced the average rate. The I^2^ value for heterogeneity was found to be 92.5% (95% CI: 88.2–95.2%). The exclusion of most studies does not significantly influence heterogeneity, remaining approximately constant between 91.6% and 93.3%, except for the study by Carugno et al. (85.9%).

**Figure 4:**
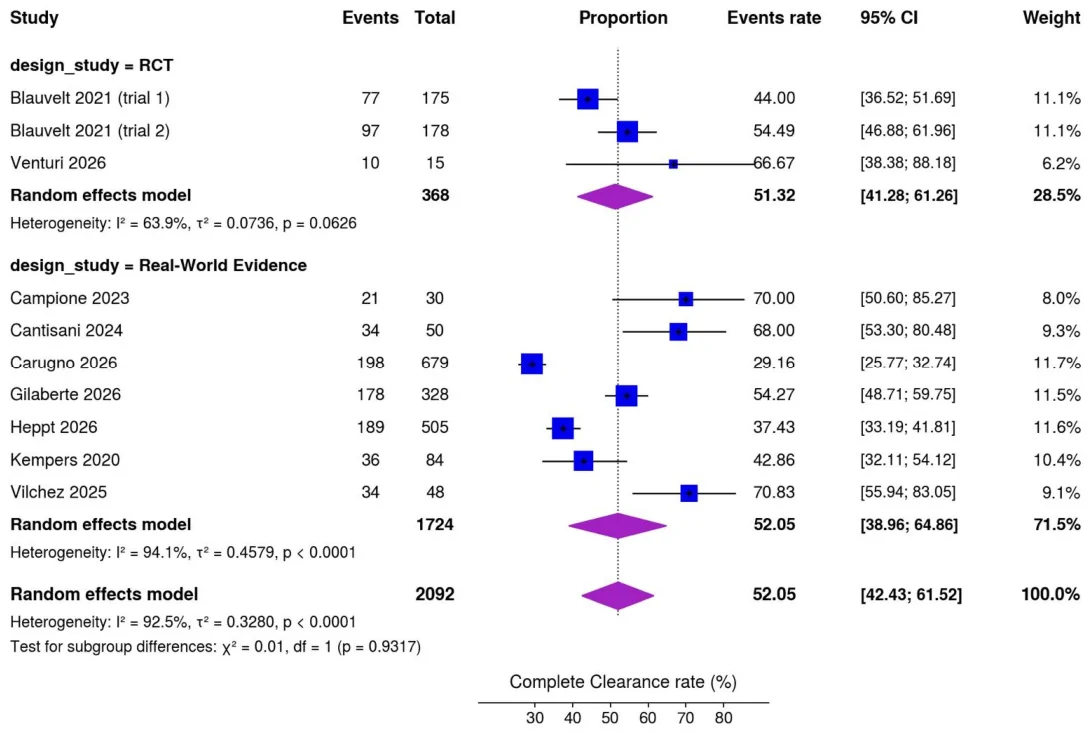
Subgroup analysis by study design of the complete clearance rate [8,9,10,11,12,13,14,16,17].

A subgroup analysis by study design (3 RCTs and 7 real-world studies) did not reveal significant differences between subgroups: the pooled CC rates were 51.32% (95% CI: 41.28–61.26%) for RCTs and 52.05% (95% CI: 38.96–64.86%) for real-world studies (Figure 4), with a *p* value of 0.932. Furthermore, the analysis of heterogeneity indicated significantly higher heterogeneity among real-world studies (I^2^ = 94.1%) compared to RCTs (I^2^ = 63.9%).

**Figure 5:**
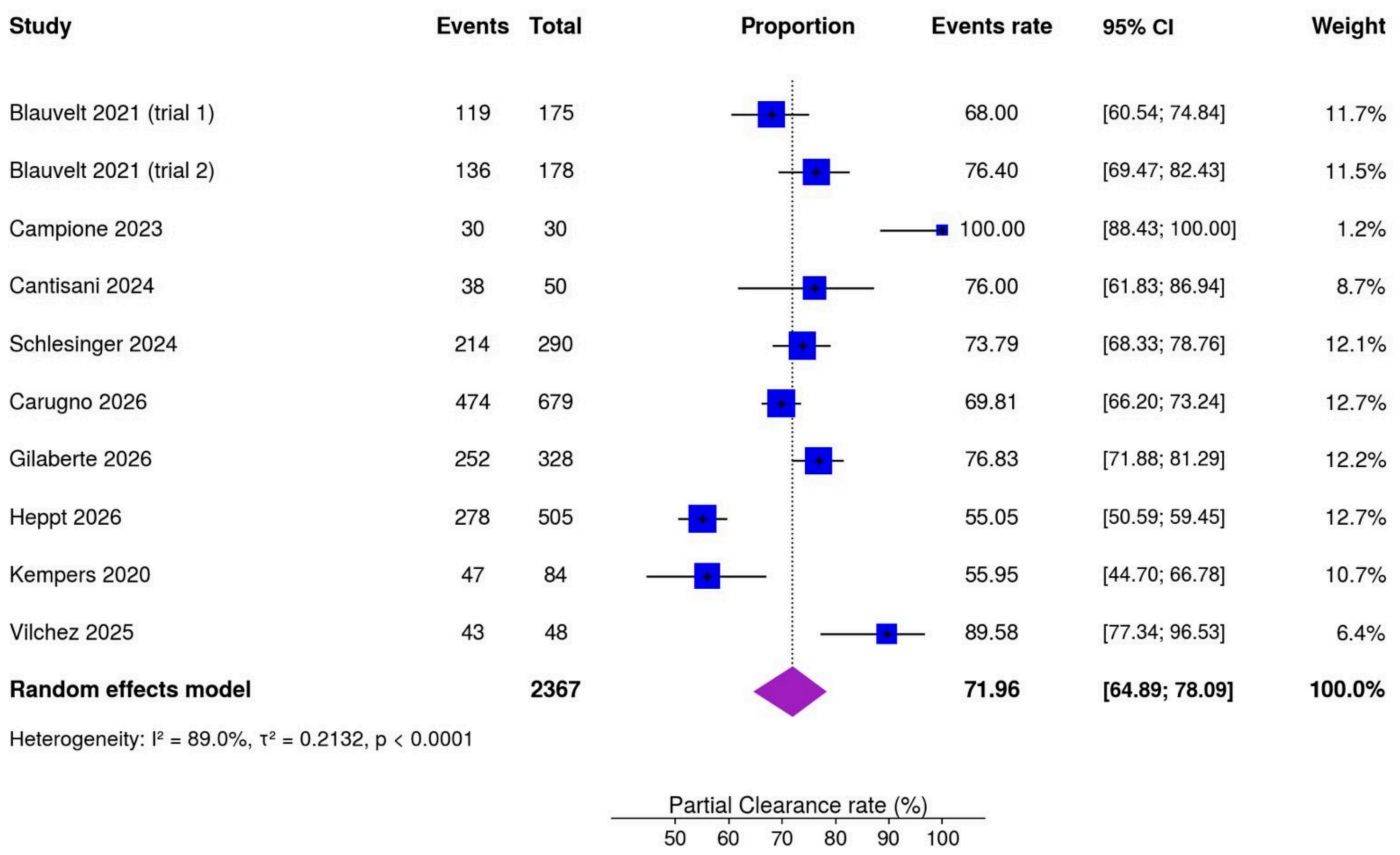
Partial clearance rates across the included studies [8,9,10,11,12,13,14,15,17].

The PC rate was reported in 10 of the 11 studies included, involving a total of 2367 patients. A total of 1631 PC events were reported, leading to an overall pooled PC rate of 71.96% (95% CI: 64.89–78.09%) (Figure 5).

**Figure 6:**
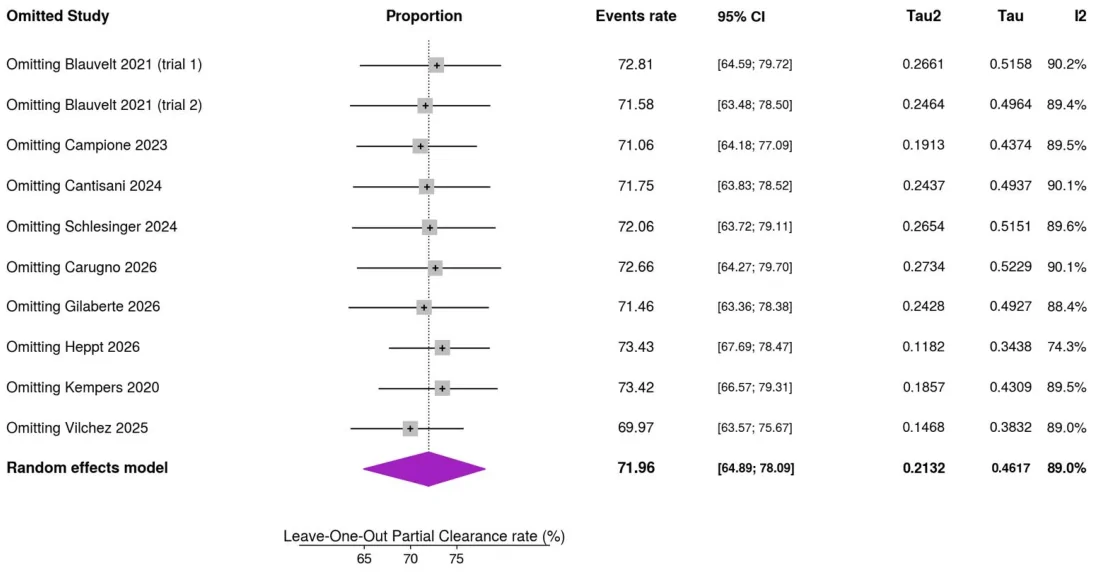
Leave-one-out sensitivity analysis for the partial clearance rate [8,9,10,11,12,13,14,15,17].

The leave-one-out sensitivity analyses showed strong robustness, with pooled PC estimates consistently falling within a narrow range of 69.97% to 73.43% after successively excluding individual studies (Figure 6). None of the studies modified substantially the average rate. The I^2^ value for heterogeneity was 89.0%. The exclusion of most studies does not significantly influence heterogeneity, remaining approximately constant between 89% and 90.2%, except for the study conducted by Heppt et al. (74.3%).

**Figure 7:**
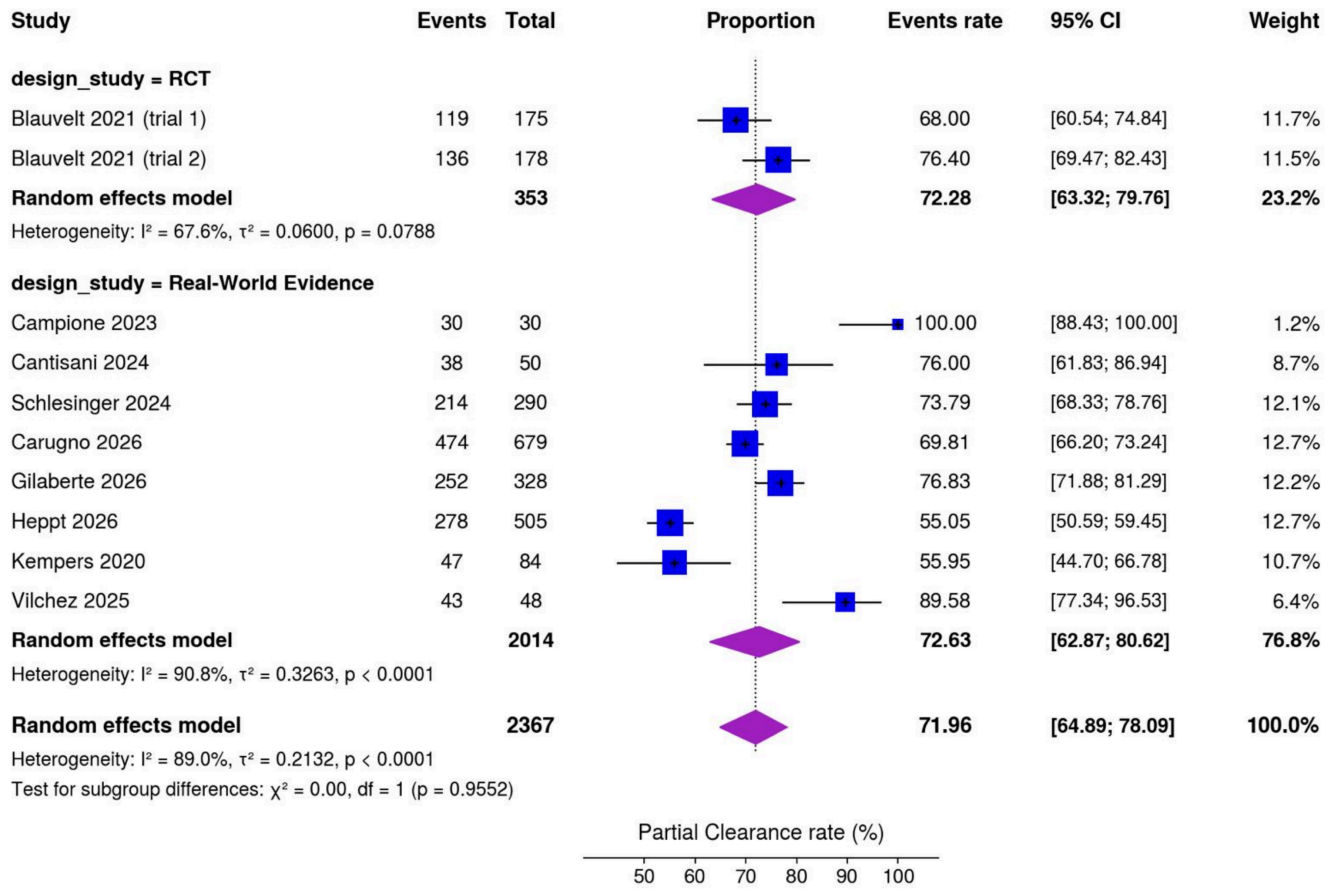
Subgroup analysis by study design of the partial clearance rate [8,9,10,11,12,13,14,15,17].

Study-design subgroup analysis (2 RCTs and 8 real-world studies) did not find significant differences between the two types of studies: pooled PC rates were 72.28% (95% CI: 63.32–79.76%) for RCTs and 72.63% (95% CI: 62.87–80.62%) for real-world studies, with a *p* value of 0.955 (Figure 7). A high heterogeneity was increased in both subgroups; however, for real-world studies, a higher I^2^ value (90.8%) was reported compared to RCT (67.6%).

**Figure 8:**
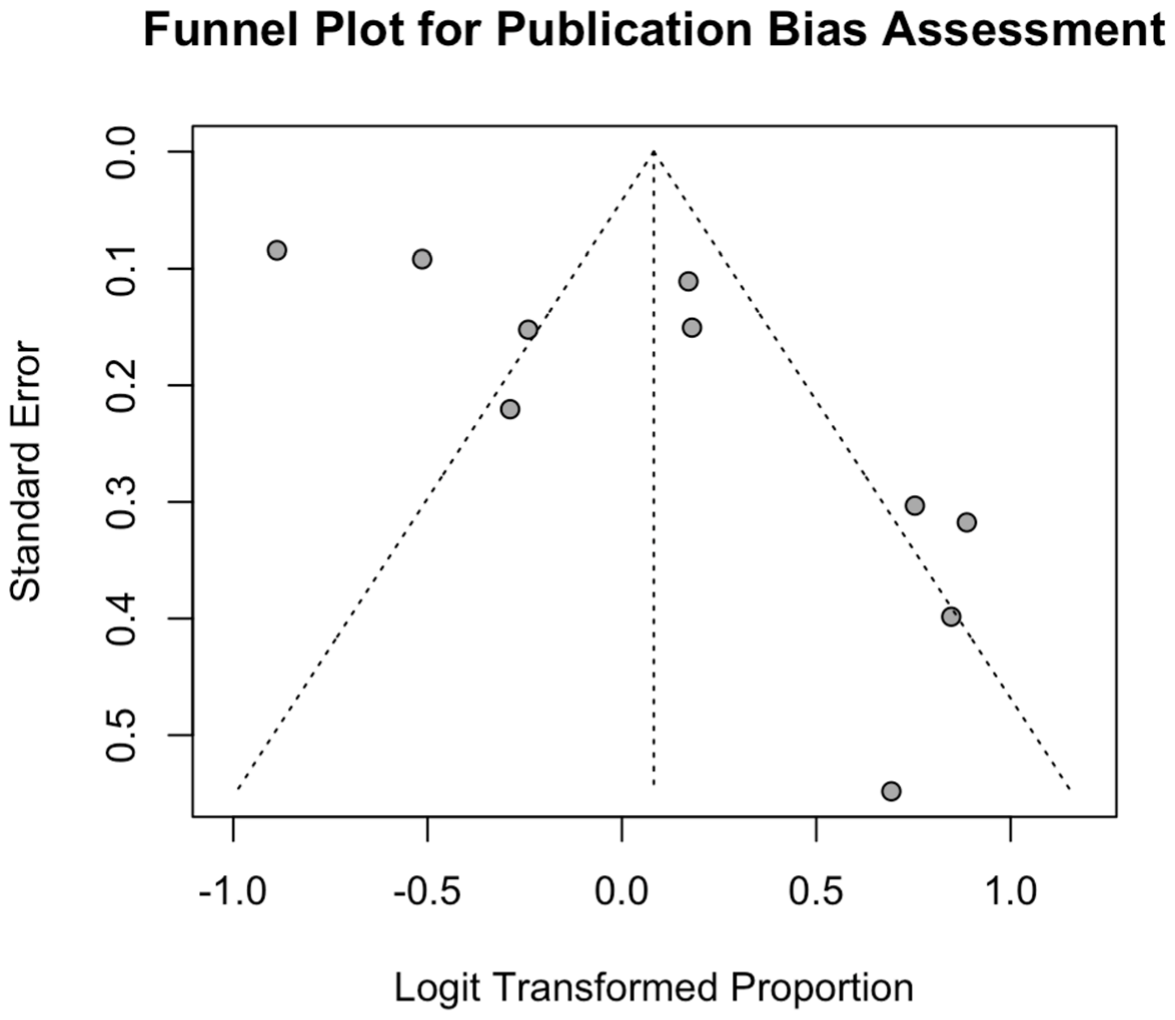
Funnel plot for the assessment of publication bias in studies reporting complete clearance rates.

Publication bias analysis for the studies evaluating CC rate revealed statistically significant funnel plot asymmetry (Figure 8). Egger’s regression test indicated *p* = 0.022, with an estimated bias of 4.97, and for Begg’s rank correlation test, *p* = 0.32.

**Figure 9:**
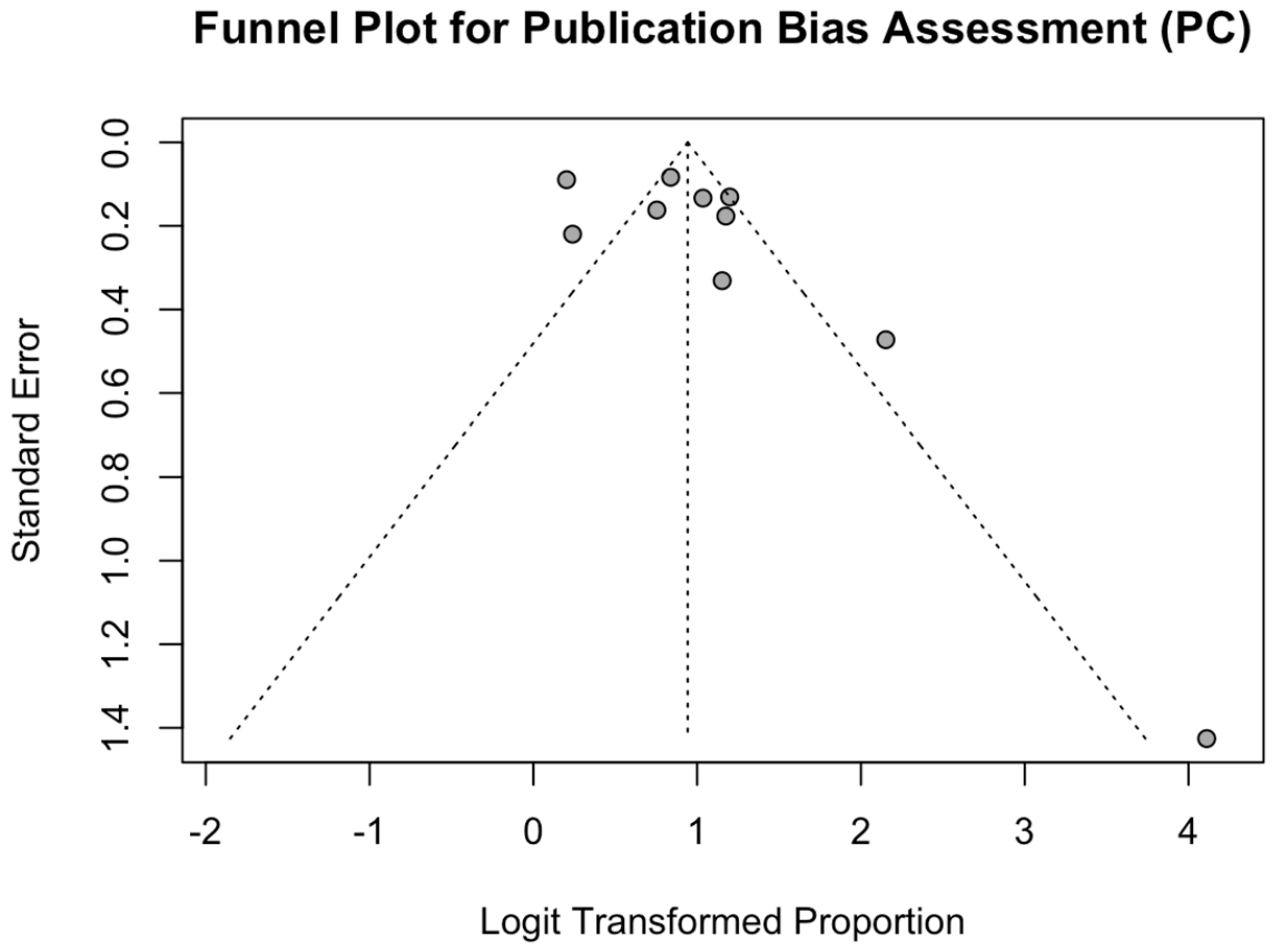
Funnel plot for assessment of publication bias in studies reporting partial clearance rates.

Publication bias analysis for the studies evaluating PC rate did not demonstrate statistically significant funnel plot asymmetry (Figure 9). Both Egger’s regression test (*p* = 0.151) and Begg’s rank correlation test (*p* = 0.420) were non-significant.

**Figure 10:**
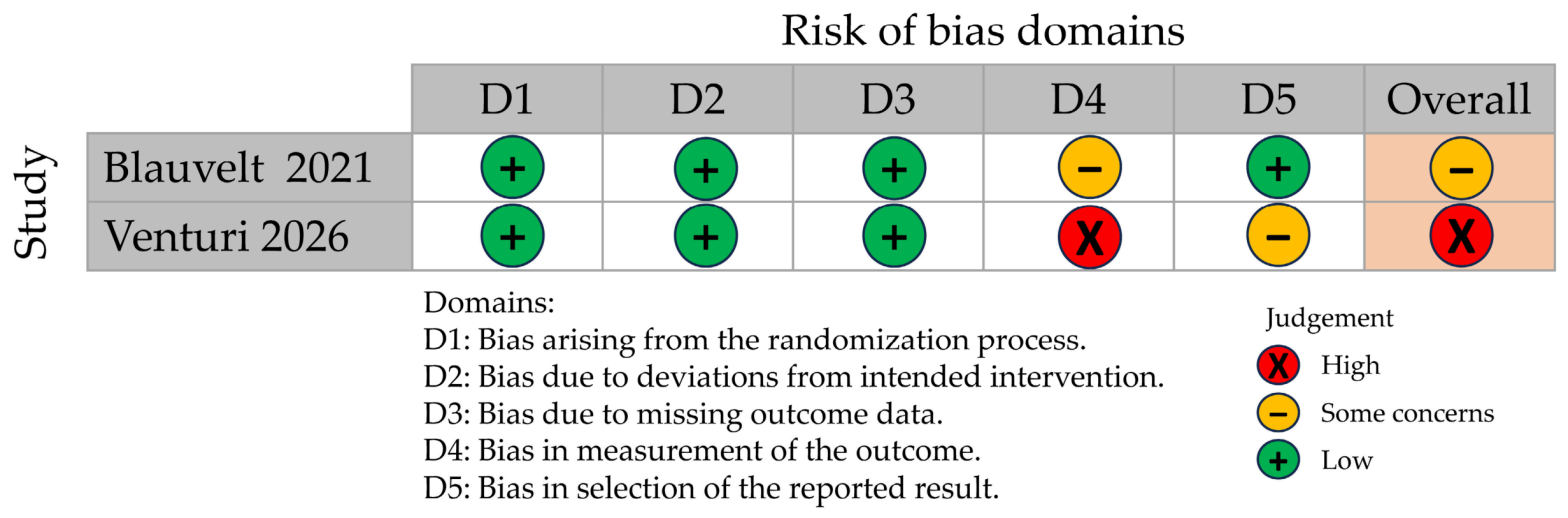
Risk-of-bias assessment of randomized studies using the revised Cochrane Risk of Bias tool for randomized trials (RoB 2) for Blauvelt et al. [8] and Venturi et al. [16]. Green indicates low risk, yellow indicates some concerns, and red indicates high risk.

**Figure 11:**
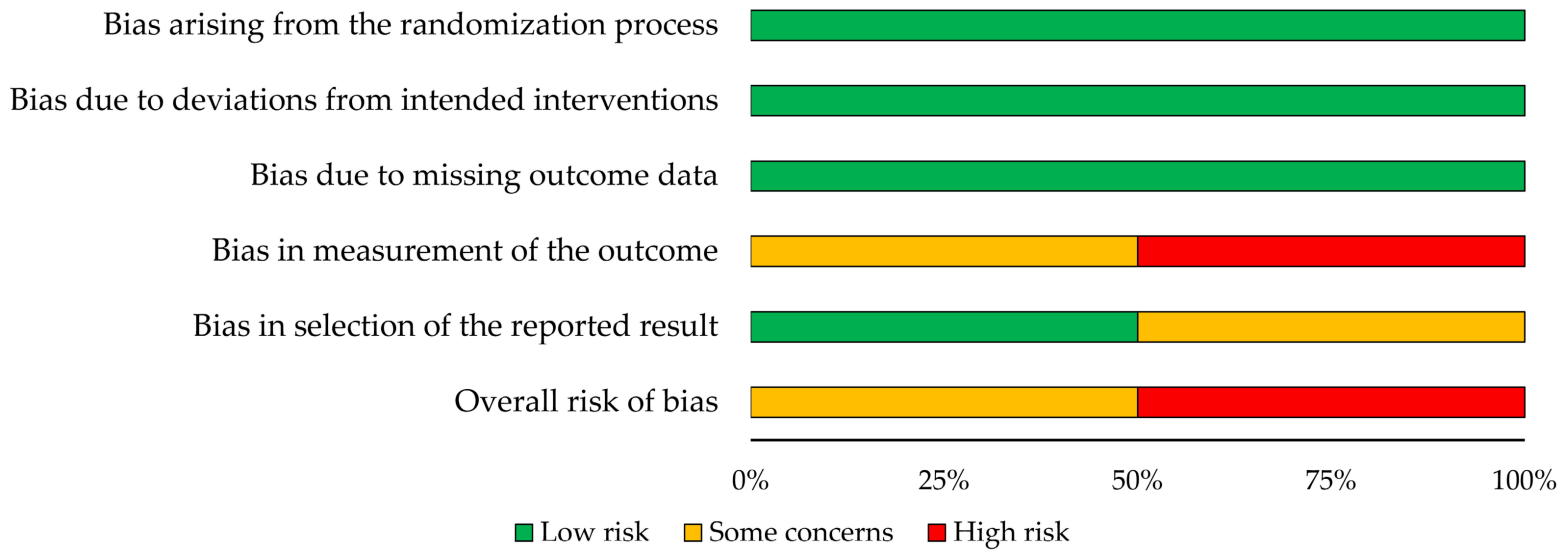
Summary plot of randomized trials presented as percentages across all included studies.

In the randomized studies, Blauvelt 2021 [8] was judged as having some concerns overall, while Venturi 2026 [16] was judged as high risk (Figure 10 and Figure 11).

**Figure 12:**
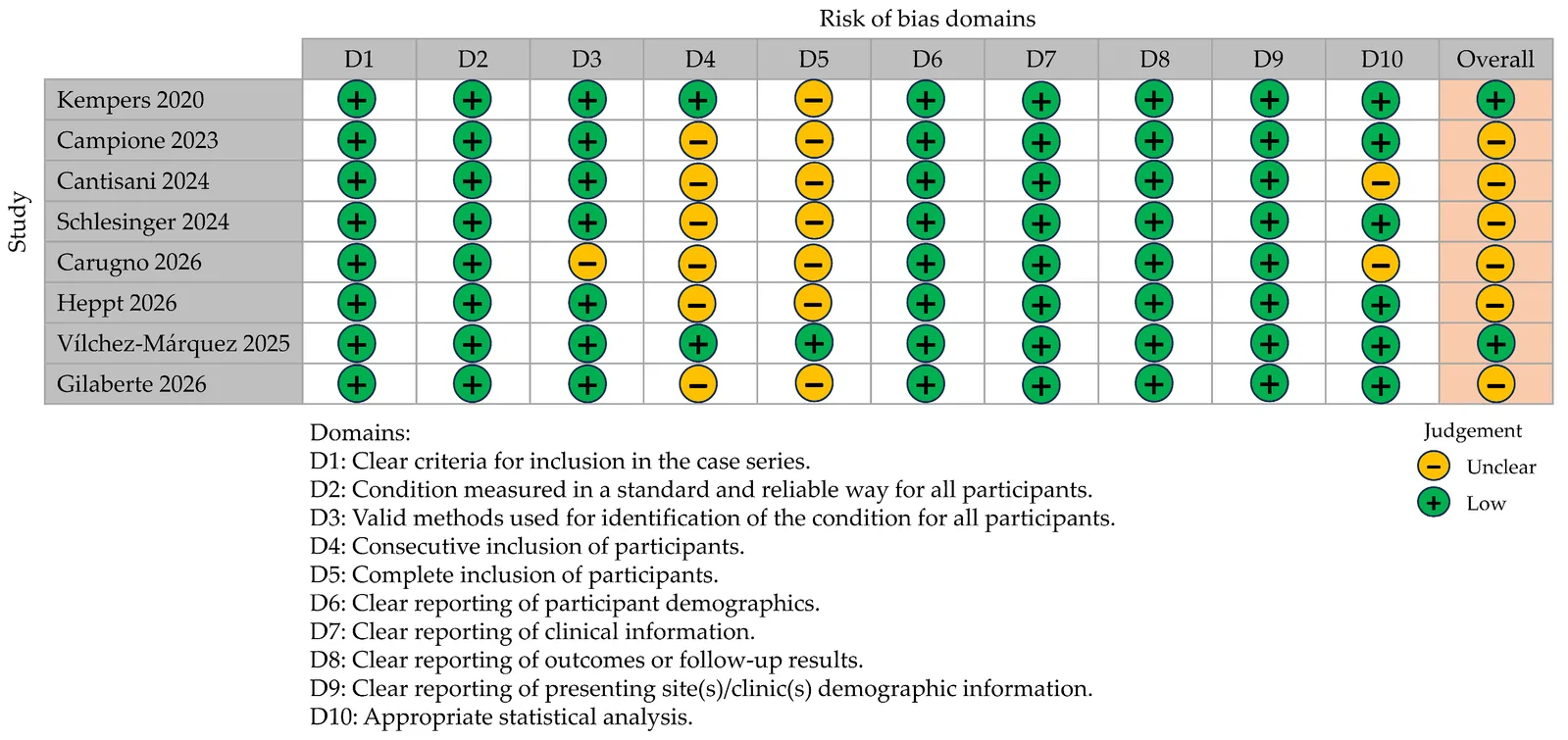
Risk-of-bias assessment of real-world studies [9,10,11,12,13,14,15,17] using the JBI Critical Appraisal Checklist for Case Series. Green indicates low risk, and yellow indicates unclear risk.

**Figure 13:**
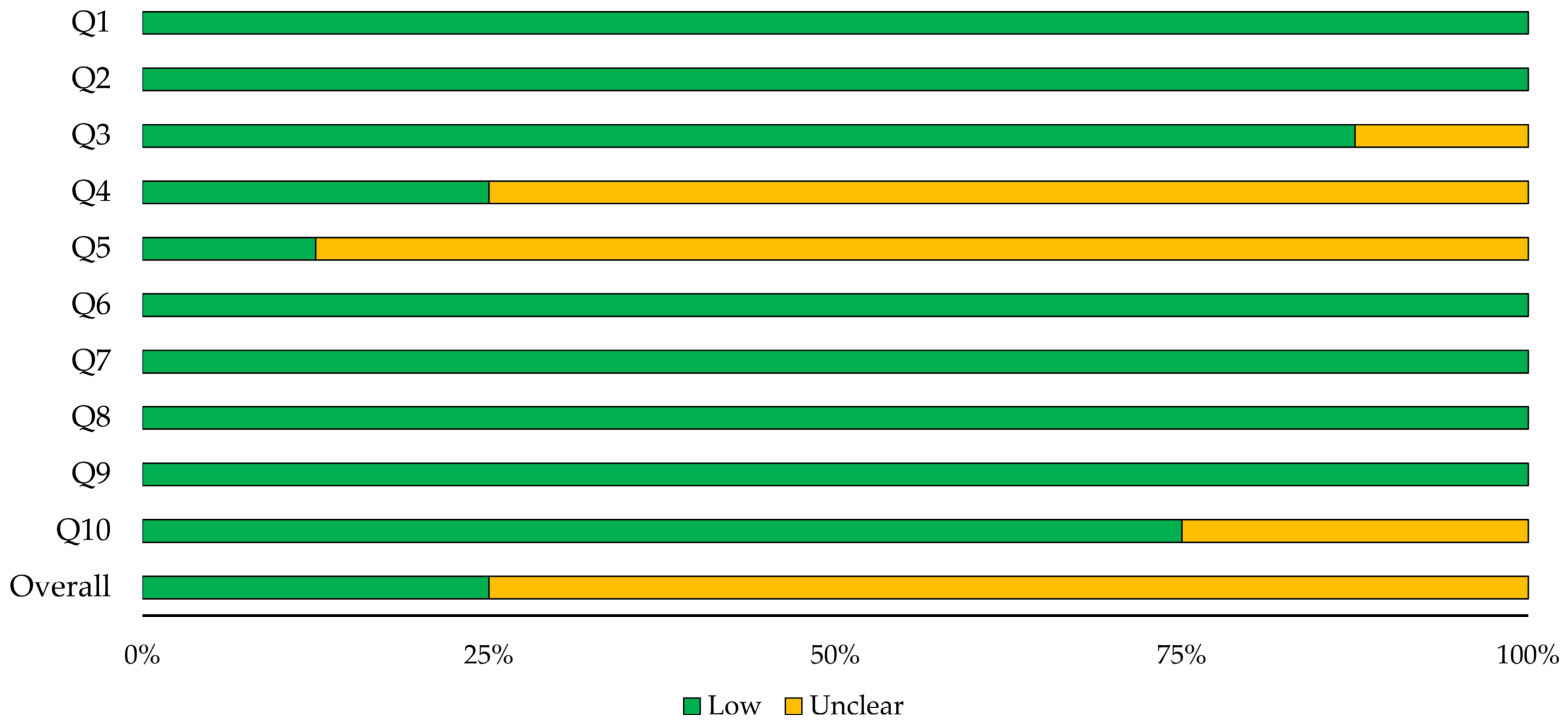
Summary plot of real-world studies presented as percentages across all included studies.

In the real-world studies, the most common methodological limitations included unclear reporting regarding the consecutive and complete inclusion of participants. Overall, Vílchez-Márquez 2025 [17] showed the most complete reporting across JBI items (Figure 12 and Figure 13).

## 4. Discussion

The primary first-line therapies for AKs include 5-fluorouracil, imiquimod, and diclofenac gel. However, these treatments have notable disadvantages, including lengthy application periods, sometimes requiring multiple applications per day [18]. These factors often lead to reduced patient compliance with treatment. A recent systematic review of 14 studies involving 4433 patients with AKs treated with topical therapies revealed that prolonged treatment time was a significant factor in lower compliance rates [19].

In this context, there is a clear need for new therapies that better meet patient needs. Tirbanibulin represents a promising option due to its short treatment protocol; therefore, it is an ideal therapy for patients with low adherence to the long-term use of topical treatments [18]. Additionally, it induces apoptosis through both intrinsic and extrinsic activation pathways, which is associated with a lower inflammatory response compared to other therapies such as 5-fluorouracil [5,20]. Moreover, tirbanibulin’s effect on microtubules is reversible, unlike classical therapies. This characteristic is associated with reduced toxicity while maintaining its effectiveness. Classic therapies such as 5-fluorouracil, imiquimod, and diclofenac require long-term treatment that lasts several weeks. It should be mentioned that these treatments can cause side effects, including erythema, itching, and the development of erosions that may, in some cases, progress to ulcers. These side effects lead to decreased adherence to the treatment in clinical practice. 5-Fluorouracil is administered twice a day over a period of approximately one month. According to the literature, the complete clearance rate is highly variable, ranging from 38% to 84%. It is important to note that the recurrence rate exceeds 60%. For imiquimod 5%, when administered twice daily for up to 16 weeks, the reported complete clearance rate is between 20% and 45%. Regarding diclofenac, treatment can be extended for up to 3 months; however, its effectiveness may be reduced in many cases [5,20].

Another notable effect of tirbanibulin is the downregulation of the Src pathway. Src family kinases (SFKs) play a key role in physiological processes in the epidermis by modulating the differentiation, proliferation, and adhesion of keratinocytes. Increased expression of SFKs is linked to disruptions in epidermal homeostasis, leading to uncontrolled keratinocyte proliferation and altered intercellular adhesion, both of which can contribute to carcinogenesis [21]. Research has indicated that elevated SFK expression is present in both AKs and in situ or invasive squamous cell carcinoma [22].

In this meta-analysis, we examined the effectiveness of tirbanibulin 1% ointment in treating AKs. We obtained a CC rate of over 50% and a PC rate of over 70% among approximately 2000 patients analyzed. The sensitivity analysis further supports these findings, indicating robustness. A comparative analysis of the two study-design subgroups—RCTs and real-world studies—did not reveal any statistically significant differences regarding effectiveness. This lack of variance may be attributed to the simplicity of the treatment protocol, which involves administering tirbanibulin 1% ointment for 5 consecutive days, which significantly reduces the risk of patients not following their treatment plans.

However, we noted increased heterogeneity in both CC and PC rates. This variability suggests differences between the samples studied, likely due to the varying designs of the included studies. We observed greater heterogeneity in real-world studies than in RCTs. To address this, we conducted a leave-one-out sensitivity analysis, which indicated that the study conducted by Carugno et al. significantly influenced the level of heterogeneity. The study by Carugno et al. reported the lowest rate of CC (29.16%). Excluding this study reduced the I^2^ statistic from 92.5% to 85.9% and increased the pooled CC rate to 54.67%. Excluding any other study had minimal effects on heterogeneity.

Similar observations were made when analyzing studies that assessed PC events. The study by Heppt et al. was the primary contributor to heterogeneity in this analysis. Its removal led to a reduction in the I^2^ statistic from 89% to 74.3%, slightly impacting the pooled PC rate, which remained at 73.43% (Heppt et al. reported the smallest PC rate—55.05%).

The funnel plot analysis exhibited different patterns for the two examined endpoints, CC and PC rates. The asymmetric pattern observed for CC rate may result from the inclusion of small studies that have low statistical power and high variability. In contrast, for the PC rate, small studies tend to show results that align closely with those of larger studies, leading to a symmetric distribution because the endpoint is less rigorous. These observations suggest there is no evidence of substantial publication bias, and the presence of small studies does not significantly impact the results reported in the existing literature. The methodological limitations identified were related to the study design rather than to the evaluation of the outcome, especially in the case of real-world studies, where the main methodological issue is related to the consecutive and complete inclusion of participants, which makes it difficult to assess the inclusion of all eligible patients.

In this meta-analysis, we included only studies that reported the patient clearance rate. However, there are several studies in the literature that have examined the rates of CC and PC in relation to the number of lesions, rather than the number of patients. A phase 3 study conducted by Bhatia et al. supports the effectiveness of tirbanibulin 1% ointment for AKs. This study included 105 patients with a variable number of AKs (ranging from 4 to 12) and analyzed the number of lesions that resolved after therapy, indicating a 77.8% reduction in the number of AKs by day 57 on a treatment field of 100 cm^2^ [23]. Similarly, Li Pomi et al. conducted a real-world study evaluating the effectiveness of tirbanibulin 1% ointment for AKs, also considering the number of AKs. This research included 38 patients with a total of 228 AKs. The CC rate was 51%, and the PC rate was 73% [24]. In another study including 32 patients with 51 AKs, Li Pomi et al. found a similar CC rate, 54.9% [25].

Kirchberger et al. also evaluated the efficacy of tirbanibulin in patients with AKs, but their study was not included in our meta-analysis because they used the AKASI score (CC was defined as AKASI less than 1). The study involved 30 patients and reported a CC rate of 57%, which aligns with the results from other studies [26].

The results of the effectiveness of topical therapies used in the treatment of AKs are variable. In 2020, Steeb et al. [27] conducted a meta-analysis to evaluate the efficacy of the most important therapeutic options for AKs. The results showed that diclofenac sodium 3% gel had the lowest complete clearance rate (23.1%), while ingenol mebutate 0.05% gel had the highest complete clearance rate (88%). Photodynamic therapy was found to have a high complete clearance rate of 74%, and imiquimod 5% cream had a complete clearance rate of 52.1%. Regarding recurrence rates, diclofenac sodium 3% gel and imiquimod 5% cream reported very high recurrence rates of 80–90%, compared to only 10–30% for ingenol mebutate gel and photodynamic therapy. These findings may be influenced by the heterogeneity of the studies analyzed, as the follow-up periods varied significantly, ranging from 36 months to shorter durations [27]. The meta-analysis by Gupta et al. evaluated the effectiveness of 22 agents used for the treatment of AKs. The findings indicated that 5-fluorouracil is the most effective agent after analyzing both complete and partial clearance. Tirbanibulin was among the 22 agents assessed, with a surface under the cumulative ranking curve (SUCRA) value of 34.79% for complete clearance [28].

In 2022, Heppt et al. [29] conducted a network meta-analysis comparing the efficacy of various topical therapies for AKs, including tirbanibulin. Their results indicated that tirbanibulin is superior to diclofenac 3% gel and has similar efficacy to photodynamic therapy, imiquimod (5%/3.75%), cryotherapy, and 5-fluorouracil (5%/4%) [29].

The recently published meta-analysis conducted by von Dannecker evaluated the efficacy and safety profile of tirbanibulin 1% ointment in patients with AKs. The meta-analysis included eight studies published up to June 2025, totaling 966 patients. They reported a CC rate of 57.6% (95% CI: 45.3–69.1) and a PC rate of 66.9% (95% CI: 50.8–79.8) and mentioned the scarce data from real-world studies as a limitation [30]. In comparison, our results showed a slightly lower rate of CC and a slightly higher rate of PC. Our meta-analysis includes a higher number of real-world studies (published after June 2025), extending the assessment of tirbanibulin effectiveness beyond the controlled conditions of RCTs, and a higher number of patients (2092 patients vs. 433 patients evaluated for CC and 2367 patients vs. 723 patients evaluated for PC). Real-world studies can complement the evidence obtained from RCTs by encompassing a broader and more diverse range of patient populations. These studies reflect routine clinical practices, which may involve variations in treatment implementation that are not fully represented in RCTs. Thus, the combined estimates should be viewed as a comprehensive synthesis of the available evidence. RCTs and observational studies were combined to give a thorough evaluation of tirbanibulin effectiveness in both controlled and real-world settings. In addition, we compared data from RCTs and real-world studies to highlight any differences. However, the comparative analysis between RCTs and real-world studies revealed no statistically significant differences, indicating that the effectiveness observed in controlled trial settings may also be achieved in everyday clinical practice. Recent data demonstrate the effectiveness of tirbanibulin in treating non-melanoma skin cancer (NMSC). In the study by Moore et al., 9 patients with SCC, 17 with SCC in situ, and 4 with basal cell carcinoma (BCC) were included. Tumor regression was observed in 80% of the cases. The treatment protocol consisted of a variable number of 5-day consecutive cycles with tirbanibulin 1% ointment, ranging from 1 to 7 cycles. It is important to interpret these results with caution, as 17 of the cases involved SCC in situ, which can be classified as AK [31].

*Limitations.* The main limitations of our meta-analysis include the high heterogeneity among the studies analyzed and the limited number of RCTs available; real-world studies were more prevalent. The increased heterogeneity can be attributed to several factors. Studies have shown differences in patient characteristics (age, gender, and immune status). The studies included in the meta-analysis varied in design; some were RCTs in phase II or III, while others were prospective or retrospective observational studies. Additionally, the number of participants in these studies varied significantly, ranging from 15 to 679. Regarding follow-up duration, most studies had an average follow-up period of 57 days, while other studies reported durations of 90 days, 12 weeks, or 24 weeks. In terms of the number of lesions treated, no significant differences were found where data were available, with the number of lesions ranging from 2 to 8. The patient groups were heterogeneous regarding prior treatments, with varying proportions of treatment-naive participants and those who had not responded to previous treatments. Among the studies that provided treatment history, the proportion of previously treated patients varied, ranging from 54.9% to 100%. The previous therapies included a range of options such as cryotherapy, PDT, diclofenac, piroxicam, 5-fluorouracil, imiquimod, and others. The data are contradictory regarding the influence of the patient’s status on the treatment response rate. Vílchez-Márquez et al. [17] reported that patients who had undergone multiple treatments had a lower response rate than treatment-naïve patients. In contrast, Schlesinger et al. observed no significant differences in response rates based on treatment history [15]. Furthermore, the studies did not utilize a standardized method for assessing the clearance of AKs, relying solely on clinical assessments that depend on the physician’s judgment.

These variables were reported inconsistently across studies, limiting additional subgroup analyses and preventing reliable meta-regression. Considering the limitations mentioned above, it is important to interpret the results obtained with caution.

The meta-analysis protocol was not registered in PROSPERO or any other registry.

## 5. Conclusions

In conclusion, although the available evidence supports the clinical effectiveness of tirbanibulin 1% ointment in treating AKs, the pooled estimates should be interpreted with caution because of the considerable variability among the included studies. Further studies are needed to include standardized tools for assessing the clearance rate.

## Figures and Tables

**Table 1:** Characteristics of the patients included in the studies.

Author	Study Design	No. of Patients	Gender	Mean Age (Years)	AK Localization	Olsen Grade	Follow-Up Period	Number of AKs	History of Previous Treatment
Blauvelt et al., 2021 (Trial 1) [8]	phase 3 multicenter RCT	175	84% male	69.5 ± 8.6	face (68%) scalp (32%)	grade I/II	day 57	4–8 lesions mean: 6	145/175 (83%) cryosurgery (63–75%) and field treatment (31–38%).
Blauvelt et al., 2021 (Trial 2) [8]	phase 3 multicenter RCT	178	89% male	69.1 ± 8.7	face (67%) scalp (33%)	grade I/II	day 57	4–8 lesions mean: 6	132/178 (74%) cryosurgery (63–75%) and field treatment (31–38%).
Campione et al., 2023 [9]	prospective observational study	30	60% male	75.3 ± 8	scalp (53.3%), face (46.7%)	grade I	day 57	2–8 lesions	26/30 (86.7%) piroxicam (27%), cryotherapy (24%), diclofenac (24%), PDT (19%), imiquimod (15%), and 5-FU (16%).
Cantisani et al., 2024 [10]	retrospective observational study	50	54% male	76 ± 7.9	face (58%), scalp (42%)	grade I	day 57	2–8 lesions	N/A
Carugno et al., 2026 [11]	retrospective multicenter study (SUMM-AK)	679	70.8% male	74.9 ± 9.7	face (61.8%), scalp (31.8%)	N/A	N/A	N/A	N/A
Gilaberte et al., 2026 [12]	phase 4 multicenter study (TIRBASKIN)	328	83.5% male	74.8 ± 8.6	scalp (52.7%), face (47.3%)	grade I/II	day 57	4–8 lesions; mean: 5.9	202/328 (61.6%) cryotherapy (43.0%), PDT (21.0%), and diclofenac (14.0%).
Heppt et al., 2026 [13]	prospective multicenter study (KLIR)	505	67.8% male	74	face (56.3%), scalp (24.7%), both (19%)	grade I	day 57	mean: 5.9	298/505 (54.9%) topical medical treatments (29.1%), cryotherapy (23.8%), PDT (8.3%).
Kempers et al., 2020 [14]	open-label phase 2 study	84	90% male	69.0 ± 8.9	face (52%), scalp (48%)	N/A	day 57	4–8 lesions; mean: 5.6	N/A
Schlesinger et al., 2024 [15]	prospective observational study (PROAK)	290	68.6% male	66.3 ± 11.4	face (77.9%), scalp	N/A	24 weeks	N/A	229/290 (79.0%) cryosurgery and other topical agents.
Venturi et al., 2026 [16]	randomized comparative study	15	73.3% male	65.5 ± 6.2	scalp	N/A	12 weeks	mean: 6.1	15/15 (100%) Diclofenac (100%)
Vílchez-Márquez et al., 2025 [17]	retrospective study	48	72.9% male	72.9 ± 10.4	scalp (58.3%), face (41.7%)	grade I	day 90	N/A	33/48 (68.8%) cryotherapy, diclofenac, 5-FU, imiquimod, or PDT.

RCT—randomized controlled trial, AK—actinic keratosis, PDT—photodynamic therapy, 5-FU—5-fluorouracil, N/A—not available.

## Data Availability

All data are contained within the article.

## Supplementary Materials

The following supporting information can be downloaded at: https://www.mdpi.com/article/10.3390/life16081330/s1, Database search strategy.
