# Supplementary material for: Clinical Clearance of Actinic Keratoses with Tirbanibulin 1% Ointment: Evidence from a Meta-Analysis
Source: Life (Basel). 2026 Aug 13;16(8):1330. doi: 10.3390/life16081330 (PMC13514861; doi:10.3390/life16081330)
Supplement: Supplementary file 1 [file life-16-01330-s001.zip › life-4455674-supplementary.pdf]

The Search Strategy

The search strategy was conducted by browsing four databases: PubMed (MEDLINE), Scopus, Web of Science, and Google Scholar (from database inception to May 28, 2026). There were no restrictions on the language or period of publication. We used MeSH terms and free keywords, including ("tirbanibulin" OR "Klisyri") AND ("actinic keratosis" OR "solar keratosis").

The complete database-specific search strategies are presented below.

PubMed/MEDLINE

("tirbanibulin"[tiab] OR "Klisyri"[tiab]) AND ("Actinic Keratosis"[Mesh] OR "actinic keratosis"[tiab] OR "actinic keratoses"[tiab] OR "solar keratosis"[tiab] OR "solar keratoses"[tiab])

Scopus

TITLE-ABS-KEY ("tirbanibulin" OR "Klisyri") AND TITLE-ABS-KEY ("actinic kerato-sis" OR "actinic keratoses" OR "solar keratosis" OR "solar keratoses")

Web of Science Core Collection

TS=("tirbanibulin" OR "Klisyri") AND TS=("actinic keratosis" OR "actinic keratoses" OR "solar keratosis" OR "solar keratoses")

Google Scholar

("tirbanibulin" OR "Klisyri") AND ("actinic keratosis" OR "actinic keratoses" OR "so-lar keratosis" OR "solar keratoses")
